# Anxiety, depression and psychosocial needs are the most frequent concerns reported by patients: preliminary results of a comparative explorative analysis of two hospital-based palliative care teams in Germany and Japan

**DOI:** 10.1007/s00702-020-02186-y

**Published:** 2020-05-17

**Authors:** Birgitt van Oorschot, Koji Ishii, Yuko Kusomoto, Lea Overbeck, Theresa Zetzl, Carmen Roch, Andreas Mettenleiter, Hiroko Ozawa, Michael Flentje

**Affiliations:** 1 Interdisciplinary Centre Palliative Care, Department of Radiation Oncology, University Hospital Würzburg, Würzburg, Germany; 2 Palliative Care Team, University Hospital Nagasaki, Nagasaki, Japan; 3 Institute of the History of Medicine, University of Würzburg, Würzburg, Germany; 4 Department of Neuropsychiatry, Graduate School of Biomedical Sciences, Nagasaki University, Nagasaki, Japan

**Keywords:** Hospital-based palliative care, Patient-reported outcome, Quaility indicator, Anxiety, Depression

## Abstract

In the partnership between the medical departments of Würzburg University, Germany, and Nagasaki University, Japan, palliative care is a relevant topic. The aim of the study was to perform a comparative analysis of the hospital-based palliative care teams in Würzburg (PCT-W) and Nagasaki (PCT-N). Survey of staff composition and retrospective analysis of PCT patient charts in both PCTs were conducted. Patients self-assessed their symptoms in PCT-W and in Radiation Oncology Würzburg (RO-W). The (negative) quality indicator ‘percentage of deceased hospitalised patients with PCT contact for less than 3 days before death’ (Earle in Int J Qual Health Care 17(6):505–509, 2005) was analysed. Both PCTs follow a multidisciplinary team approach. PCT-N saw 410 cancer patients versus 853 patients for PCT-W (22.8% non-cancer patients). The Eastern Cooperative Oncology Group Performance Status at first contact with PCT-N was 3 or 4 in 39.3% of patients versus 79.0% for PCT-W. PCT-N was engaged in co-management longer than PCT-W (mean 20.7 days, range 1–102 versus mean 4.9 days, range 1–48). The most frequent patient-reported psychological symptom was anxiety (family anxiety: 98.3% PCT-W and 88.7% RO-W, anxiety 97.9% PCT-W and 85.9% RO-W), followed by depression (98.2% PCT-W and 80.3% RO-W). In 14 of the 148 deceased patients, PCT-N contact was initiated less than 3 days before death (9.4%) versus 121 of the 729 deceased PCT-W patients (16.6%). Psychological needs are highly relevant in both Germany and Japan, with more than 85% anxiety and depression in patients in the Japanese IPOS validation study (Sakurai in Jpn J Clin Oncol 49(3):257–262, 2019). This should be taken into account when implementing PCTs.

## Introduction

As part of joint research activities between the medical departments of Nagasaki University and Würzburg University that started in 2011, our focus was set on palliative care. In the spirit and tradition of the famous Würzburg-born physician and Japanologist Philipp Franz von Siebold (1796–1866) who used his stay in Nagasaki between 1823 and 1829 to teach Japanese colleagues about Western medicine and to learn about Japanese science and culture, a fruitful exchange of practical and personal experience was made possible by visiting the palliative care units in both university hospitals and sharing opinions as well as knowledge gained from our respective experiences.

A closer look at the facts and figures revealed similarities as well as differences, and this comparison might be a useful new starting point for reflecting upon ‘traditional’, possibly entrenched, convictions and for discussing practical consequences of or within the respective existing systems, especially as those ‘grown structures’ are the result of both different historical developments and cultural backgrounds that can be evaluated better by considering the outcome of different solutions to similar problems in different countries.

It is widely accepted that palliative care was not ‘invented’ by the hospice movement in the 1960s (Stolberg 2013/2017) and put into practice for the first time in the 1980s in palliative care units, but that its roots date back much farther. This can be easily shown in Würzburg: inspired by Italian Renaissance hospitals, the catholic Julius Hospital took care of the physical as well as the spiritual side of its patients—visiting the sick was considered one of the Christian works of mercy. The fact that clearly incurable persons were not accepted for treatment to use the limited resources for healing curable patients is not a contradiction: written instructions specified how to handle and nurse dying and severely ill persons where no medical help was possible (Mettenleiter 2001). Humanistic physicians also wrote about palliative care (‘cura palliativa’). Of the Christian doctors who felt the shortcomings of a purely medical treatment and proposed a more holistic view of end-of-life treatment, the Würzburg physician August Stöhr (1843–1890), who wrote the popular *Textbook of pastoral medicine* (1878), should be mentioned.

Cancer has been the leading cause of death in Japan since 1981. About 370,000 people died in Japan from cancer in 2017. After heart failure, cancer is the second most frequent cause of death in Germany. In Germany, about 235,700 people died of cancer in 2017. Regarding the history of modern palliative care, it was especially initiated in the case of cancer patients and their families. Multidisciplinary non-profit societies for palliative medicine were funded in Germany in 1994 and in Japan in 1996. In the last few decades, great efforts have been made in both countries to improve palliative care starting with initiatives to improve palliative care especially for cancer patients nearing end of life. Several laws and programmes supported the implementation of palliative care (Morita et al. 2013; Sakashita et al. 2018; Leitlinienprogramm Onkologie 2019).

At both University Hospital Nagasaki and University Hospital Würzburg hospital-based palliative care teams (PCTs) are well established; in University Hospital Würzburg, these are supplemented by a palliative care unit with ten beds. In 2019, an exchange about the work of the two hospital-based palliative care teams began. Hospital-based palliative care teams have a tradition going back to the 1980s, starting in the UK, the USA, Canada and Australia (Dunlop and Hockley 1999). A review and actual analysis of US PCTs has shown the benefits of PCTs for cancer patients in regard to pain management, the management of other symptoms, as well as psychosocial and health-care outcomes (Higginson Evans 2010; Schoenherr et al. 2019) also updated in the German S3-LL Palliative Care for Incurable Cancer Patients Guidelines (S3-LL Palliativ, Leitlinienprogramm Onkologie 2019, Chapter 5).

In Japan, the funding of PCTs was implemented in 2002. Structural requirements for PCTs were established in 2009, predominantly with regard to patients with cancer: a PCT must have a full-time palliative care physician, psychiatrists, nurses and pharmacists. PCT meetings must be held more than once a week and a palliative outpatient clinic, palliative care consultation with community health-care providers and discharge support for hospitalised patients must be offered (Sasahara et al. 2009). In 2018, a Palliative Care Consultation Team Standard was developed using a modified Delphi method with the intention to adopt this standard in all PCTs in designated cancer hospitals by the end of 2020 (Sakashita et al. 2018).

In Germany, hospital-based PCTs have been financed by health insurance funds since 2008. The funding is linked to the fulfilment of several structural requirements: an autonomous team with a palliative care physician, a palliative care qualified nurse and at least one team member with a ‘third profession’, either a psychologist, social worker or physiotherapist/occupational therapist. A detailed assessment of symptoms and needs, an individual multidisciplinary therapy plan and weekly team meetings must be carried out for each patient. The S3-LL Guidelines contain further recommendations: regular evaluation of the interventions, support for and close cooperation with the primary team and frequent interaction with outpatient-based physicians and carers, especially with specialised outpatient palliative care teams, hospices and primary physicians (Leitlinienprogramm Onkologie 2019, Chapter 5, 5.3.1). Palliative care units and palliative care teams are considered to be supplemental offerings (Gaertner et al. 2012).

The German S3-LL Guidelines also contain recommendations regarding symptom control and structural requirements. A standard operation procedure (SOP) is recommended for the demand-oriented integration of multidisciplinary specialised palliative care into cancer patients’ medical care, based on the complexity of care regarding patient-reported needs, problems with care, patients’ general conditions and the Australian casemix classification for palliative care (Eagar et al. 2004). To assess the needs of patients and relatives, standardised symptom checklists such as the Edmonton Symptom Assessment Scale (ESAS, Bruera 1991) or the Palliative care Outcome Scale (POS, Hearn and Higginson 1999) are recommended. The use of those instruments is also recommended for German Comprehensive Cancer Centres. The IPOS addresses what patients report as their main concerns (Murtagh and Ramsenthaler 2019). The revised version of the POS, the integrated POS (IPOS), is validated in Japanese as well as in German (Murtagh and Ramsenthaler 2019; Sakurai et al. 2019).

A first glance at the everyday life of the PCTs in Würzburg and Nagasaki suggested that the working principles and patients’ needs seemed similar. Therefore, we evaluated the feasibility of a comparative analysis of the co-management of PCT Würzburg (PCT-W) and PCT Nagasaki (PCT-N) with a special focus on team composition and working mode, the clinical characteristics of patients, symptom burden and contents of care, and on the integration of the PCTs into end-of-life care in the two hospitals. Because only PCT-W assessed patient-reported concern, in the discussion the Würzburg data are compared to the data from the Japanese validation study (Sakurai et al. 2019) in the discussion.

## Methods

In the third expert meeting in October 2019 in Würzburg, the composition and the working mode of the two teams were documented. The charts of PCTs’ patients until December 2019 were analysed regarding patient characteristics and contents of care in 2018, as routinely documented in both PCTs. Since January 2019, the IPOS has been established in PCT-W as well as in the routine symptom assessment of all cancer patients in the waiting time before first contact with the physician in radiation oncology. Symptom burden and needs assessed with the IPOS between January and September 2019 were analysed retrospectively and anonymously using PCT-W’s patient charts and the self-assessment questionnaires of all adult cancer patients in the Department of Radiation Oncology (regardless of the intention to treat ‘curatively’ or ‘palliatively’).

The IPOS integrates the most important patient-reported symptoms and concerns in a 17-item multidimensional tool examining physical and psychiatric symptoms as well as communication, spiritual and practical issues (Higginson et al. 2012). Answers are given on a 5-point Likert scale (0 = not at all, 1 = slight, 2 = moderate, 3 = severe, 4 = overwhelming, Murtagh and Ramsenthaler 2019). In comparison with Sakurai et al. (2019), symptoms scored ‘slight’ or higher were defined as clinically relevant.

To assess the integration of specialised palliative care into the end-of-life care of hospitalised cancer patients, two well-established quality indicators were used: (1) ‘percentage of deceased hospitalised patients with PCT contact’ and (2) ‘percentage of patients with PCT contact for less than 3 days before death’ were analysed. These quality indicators were first presented by Earle et al. for benchmarking. They can be assessed very easily using the charts of the deceased hospitalised patients. The proposed expert-approved quality goals are 55% for PCT contact before death and less than 8% for PCT contact for less than 3 days before death for all patients with PCT contact (Earle et al. 2005).

## Results

### Team composition and working mode

Both PCTs follow a multidisciplinary team approach: the core team of PCT-N contains two anaesthesiologists, two psychiatrists, three registered nurses, five pharmacists and two dieticians. That of PCT-W has one anaesthesiologist, one radiation oncologist, one general practitioner, three palliative care nurses, one social worker and one psychologist. On-demand hospice volunteers and all other professionals at the two university hospitals can be involved in patients’ care.

In Würzburg, patients are assigned to the PCT within the framework of a consultation, which is initiated by the physicians of the primary teams. In Nagasaki, the PCT takes part in the medical visits on work days. After the visit, a joint decision is made as to which patients should be co-managed by the PCT. Weekly meetings with patients’ doctors and nurses take place in both Nagasaki and Würzburg. PCT-N works without a palliative care unit in the background and also gives continuous care for outpatients after discharge. PCT-W has the option of transferring patients to another palliative care unit and cooperates with specialist outpatient PCTs in the region.

### Patient characteristics

PCT-N saw 410 patients (100% cancer patients), while PCT-W saw 853 patients (22.8% of them were non-cancer patients). The mean age of the Nagasaki patients was 67 years (median 64.7) versus 69 years (median 68.4 years) for the Würzburg patients. 59.0% of Nagasaki patients and 52.8% of Würzburg patients were male. The ECOG Performance Status of the Nagasaki patients at first contact with the PCCS was 0–1 in 34.1%, 2 in 26.6%, 3 in 21.5% and 4 in 17.8% versus 0–1 in 6.1%, 2 in 14.9%, 3 in 40.8% and 4 in 38.2% for the Würzberg patients. Seventy-seven patients who died in hospital were co-managed by PCT-N (18.8%) versus 106 patients who were co-managed by PCT-W (12.4%). See Table 1: patient characteristics for more details.

**Table 1:** Patient characteristics (2018)

Item	Nagasaki	Würzburg
Number		
* n*	410	853
Age		
Median, mean	67, 64.7	69, 68.4
Gender		
Male *n*, %	242, 59.0	450, 52.8
Female *n*, %	168, 41.0	403, 47.2
Cancer patients *n*, %	403, 98.3	664, 77.8
General condition (ECOG)		
At first contact *n*, %		
ECOG 0–1	140, 34.1	42, 4.9
ECOG 2	109, 26.6	102, 12.0
ECOG 3	88, 21.5	278, 32.6
ECOG 4	73, 17.8	260, 30.5
Missing	–	171, 20.0
Duration of PCT co-management in days		
Median, mean, range	15, 20.7, 1–102	4, 4.9, 1–48
Deceased in hospital during		
PCT co-management *n*, %	77, 18.8	106, 12.4

*PCT* palliative care team

### Patient-reported symptoms and psychosocial needs

**Fig. 1:**
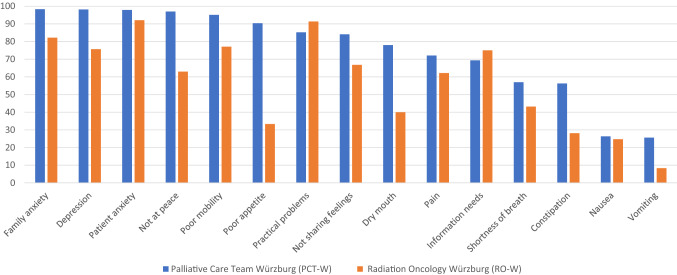
Patient-reported concerns

Between January and September 2019, 498 patients in PCT-W filled in the IPOS in its entirety. Between May to December 2018, 549 cancer patients filled in the IPOS in the University Hospital Würzburg’s Department of Radiation Oncology during the waiting time before first contact with the radiation oncologist (RO-W). 174 of them were treated with palliative intent and 375 with curative/adjuvant intent (68.3% curative, 31.7% palliative). Anxiety was the most cited symptom (family anxiety: 98.3% PCT-W and 82.2% RO-W, patient anxiety: 98.2% PCT-W and 92.1% RO-W), followed by depression (98.2% PCT-W and 75.7% RO-W), not feeling at peace (97.0% PCT-W and 63.0% RO-W), poor mobility (95.1% PCT-W and 77.1% RO-W), poor appetite (90.4% PCT-W and 33.3% RO-W), practical problems (85.2% PCT-W and 91.4% RO-W), not sharing feelings (84.1% PCT-W and 66.8% RO-W), dry mouth (78.0% PCT-W and 40.0% RO-W), pain (72.1% PCT-W and 62.2% RO-W), information needs (69.4% PCT-W and 75.0% RO-W), shortness of breath (57.0% PCT-W and 43.2% RO-W), constipation (56.3% PCT-W and 28.2% RO-W), nausea (26.3% PCT-W and 24.7% RO-W) and vomiting (25.6% PCT-W and 8.3% RO-W); see also Fig. 1: patient-reported concerns.

### Contents of care

PCT-N was engaged in palliative care co-management for longer than PCT-W (mean 20.7 days, median 15 days, range 1–102 days versus mean 4.9 days, median 4.0 days, range 1–48 days; see Table 2: contents of care). PCT-N was involved in symptom control in 95.8% of patients versus 39.8% in Würzburg. In 30.5% of cases, PCT-W was involved because of questions regarding transferral to a palliative care unit—either in-house or close to the patient’s home. Coordination of outpatient palliative care was done by PCT-N in 45.4% (186 of the 410 patients) and by PCT-W in 19.9% of patients. For more details, see Table 2: contents of care.

**Table 2:** Contents of care and outcomes in 2018, *n* = 410 patients in Nagasaki and *n* = 853 patients in Würzburg, multiple responses possible

Item	Nagasaki	Würzburg
Symptom control	393 (95.8%)	340 (39.8%)
Support in therapy goal finding	18 (4.4%)	64 (7.5%)
Advanced care planning	5 (1.2%)	–
Family consulting and support	33 (8.0%)	12 (1.4%)
Coordination of outpatient palliative care	186 (45.4%)	162 (18.9%)
Admission to palliative care unit	–	261 (30.5%)
Support of dying patients, their relatives and team	35 (8.5%)	106 (12.4%)
Information about palliative care	3 (0.7%)	–

### Integration of specialised palliative care (SPC) into inpatient end-of-life care

**Fig. 2:**
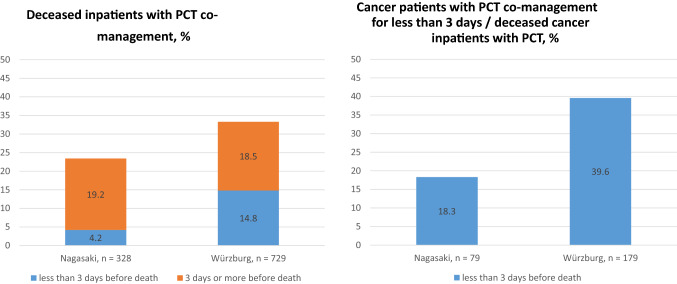
Integration of SPC into inpatient end-of-life care

18.8% of Nagasaki patients died during PCT co-management versus 12.4% of Würzburg patients. 23.5% of all deceased patients in University Hospital Nagasaki had contact with the PCT versus 35.1% of deceased patients in University Hospital Würzburg. In 14 of the 148 deceased patients with PCT contact in Nagasaki, the palliative co-management was initiated less than 3 days before death (9.4%) versus 121 of the 729 deceased PCT patients in Würzburg (16.6%). If only the cancer patients are considered, there were notably more deceased patients co-managed by SPC (18.3% in Nagasaki and 39.6% in Würzburg; for more details see Fig. 2: integration of SPC into inpatient end-of-life care).

## Discussion

To our knowledge, this is the first comparative analysis of team composition and daily work of a Japanese and a German PCT. A retrospective analysis of patients’ charts regarding two quality indicators to evaluate the integration of a PCT into inpatient end-of-life care was performed. However, the strength of this explorative analysis is also a limitation. Routine data from different cultural backgrounds were used, which were not collected for research and a previous agreement on the items to be collected was not made. This may sometimes result in fuzziness and misunderstandings cannot be ruled out.

### Team composition, working mode, patient characteristics and contents of care

Despite the different health-care settings, the national requirements for team structure are similar. It is therefore not surprising that the team compositions of the two PCTs resemble one another. Regarding German requirements, the involvement of psychiatrists in a PCT is a special feature. The PCT-N patients in general had a better general condition than the PCT-W patients and the duration of PCT-N co-management was longer. It remains unclear whether this was due to Nagasaki patients having earlier PCT contact or to a longer inpatient stay. The German study of Erlenwein et al. has shown that the PCT was contacted about 6–8 days after admission to hospital with a mean hospitalisation of 13–14 days (0–36 days in surgical departments, and 0–138 days in non-surgical departments, Erlenwein et al. 2014). In the study of Schoenherr et al., PCT consultations were requested a mean of 4.8 days into the hospital stay (range 1.7–11.1 days, Schoenherr et al. 2019). In Nagasaki the first PCT contact was earlier and also initiated for patients in better general condition, maybe due to the joint visits.

Similar differences in the general condition of Japanese and German PCT patients were found in the German study by Gaertner (70% of PCT patients with ECOG 3 or 4 in Cologne, Gaertner et al. 2012) and in the Japanese study by Sakurai (15.7% of palliative patients with ECOG 3–4 (34% outpatient clinic, 19% PCT and 46% palliative care unit, Sakurai et al. 2019). This may also be a consequence of outpatient care in the German health-care system being predominant. In Erlenwein’s study, 24% of PCT patients were transferred to a palliative care unit (Erlenwein et al. 2014), fewer than in Würzburg (30%). The range of deceased patients during PCT co-management is comparable to other studies: 20.3% in Schoenherr et al. (2019) and 12% in Gaertner et al. (2012).

It can be assumed that structural requirements and funding influence PCT co-management. PCT-N has an explicit focus on cancer patients without the option of transferring them to a palliative care unit and PCT-W can only cooperate with specialised outpatient palliative care if such a team exists close to a patient’s home. It is very interesting that despite the different team composition of the German and Japanese PCTs, psychosocial concerns dominate patient-reported symptoms and needs. Because the treatment strategies were not documented in a comparable manner, analysis was not possible. Future studies should examine these aspects in detail. Harmonisation of items and data elements for further prospective comparative analysis is a desideratum. In the Palliative Care Quality Network (PCQN) in the USA as well as in the German Hospice and Palliative Registry (Nationales Hospiz-und Palliativregister), different core sets of data for the benchmarking of PCTs were defined (Schoenherr et al. 2019; Nationales Hospiz-und Palliativregister). These could be built on.

### Symptoms and psychosocial needs

The majority of PCT-W patients reported physical, psychological, social or spiritual symptoms and needs. In a previous study performed by Körner et al. using the Edmonton Symptom Assessment Scale (ESAS, Bruera 1991) in radiation oncology, 72% of curatively and 86% of palliatively treated patients reported at least one clinically relevant physical or psychological symptom, mostly decreased general well-being (28% of curative and 62% of palliative patients), followed by tiredness (35% of curative patients) and pain (62% of palliative patients, Körner et al. 2017).

Reilly et al. performed a literature synthesis of 21 studies with a total of 4067 cancer patients receiving active treatment regarding symptom prevalence and severity. The most cited symptoms were generalised fatigue (pooled prevalence 59%), followed by insomnia/disturbed sleep (48%), pain (48%), dry mouth (47%) and anorexia/appetite changes (45%). They propose the use of a core set of symptoms that also include ‘depression’ and ‘difficulty concentrating/remembering’ for assessment across clinical trials and in the routine of cancer care, particularly among patients with advanced diseases (Reilly et al. 2013). From our point of view, the IPOS maps this core set very well, especially with its additional option of freely naming further symptoms and needs. The higher burden of the patients in Radiation Oncology Würzburg regarding information needs and practical problems (see Fig. 1) is most likely due to the time of the interview (before first contact with the physician). It would be interesting to repeat the survey again afterwards; perhaps some of the concerns were addressed during discussions with the radiation oncology team.

Over 90% of the PCT-W patients reported one or more psychiatric symptoms or psychosocial needs and more than 50% reported one or more physical symptoms. This is higher than that reported in the Japanese validation study of the IPOS (142 adult patients in palliative care units in six hospitals, Sakurai et al. 2019; for more details see Table 3: palliative concerns PCT-W and Japanese PCU patients). In both PCT-W and Sakurai’s study, the IPOS was assessed at first contact with palliative care specialists. It remains unclear whether this remarkable difference in palliative concerns is due to the different settings (palliative care unit versus PCT co-management), assessment in another phase of the disease, or to cultural or other aspects.

**Table 3:** Palliative concerns of PCT-W patients and Japanese PCU patients (PCU-J, Sakurai et al. 2019), counts in %

Item	PCT-W	PCU-J
Family anxiety	98	88.7
Depression	98.2	80.3
Anxiety	97.9	85.9
Not at peace	97.0	85.1
Poor mobility	95.1	75.4
Poor appetite	90.4	66.7
Practical problems	85.2	68.6
Not sharing feelings	84.1	70.2
Dry mouth	78.0	63.1
Pain	72.1	66.2
Information needs	69.4	57.2
Shortness of breath	57.0	53.3
Constipation	56.3	58.9

Unfortunately, further comparison is not possible, because in the international literature the threshold values for symptom prevalence are defined as any IPOS symptoms/problems specified as ‘moderate’, ‘severe’ or ‘overwhelming’ and not the ‘slight’ or higher threshold that was used by Sakurai et al. and in the Würzburg analysis. In the IPOS validation study, patients in German and British palliative care units or under PCT co-management were interviewed (392 participants, 77.7% cancer patients, Murtagh and Ramsenthaler 2019). Symptom prevalence was between 14.6% (vomiting) and 84.8% (family anxiety). As shown in the Würzburg data and in the Japanese validation study, psychosocial concerns (and weakness/poor mobility) were most frequently cited (83.5% information needs, 81.7% weakness, 77.4% poor mobility, 75.0% not sharing feelings, 72.1% not at peace, 71.0% patient anxiety). Further research is needed.

Regarding psychological symptoms and needs, 86–97% of patients in our samples reported anxiety or depression. This is considerably more than in the analysis of Reilly and colleagues (34% depression/sadness, no information given regarding anxiety, Reilly et al. 2013). In the study of Körner et al., more than 30% of patients had clinically relevant depression or anxiety before first contact with the radiation oncologist. The differences between curative and palliative patients were not significant (Körner et al. 2017). In the study of Schoenherr et al. (2019), 35% of patients reported anxiety. The difference between our data and the literature is perhaps a result of different cutoff points. In comparison with Sakurai et al., we defined the symptom cutoff as ≥ 1 in the 5-point Likert scale used in the IPOS. In the studies of Reilly et al. and Körner et al., the cutoff for clinically relevant symptoms or needs was defined as ≥ 4 in 10-point Likert scales. It also remains unclear whether the self-assessment of ‘feeling depressed’ and ‘feeling anxious or worried about your illness or treatment’ is concordant with the clinical diagnosis of a depression or anxiety disorder.

The S3-LL Guidelines recommend the use of validated screening tools for the detection of depression and anxiety such as the ultra-brief Patient Health Questionnaire for Depression and Anxiety (PHQ4, Löwe et al. 2010), followed by a detailed assessment for screening positive patients. Harriet Webler showed in her dissertation that self-assessment (measured with the ESAS items ‘depression’ and ‘anxiety’) only matched with PHQ4 (unpublished data) in about 40% of cases. The S3-LL Guidelines contain detailed chapters on anxiety and depression and the European Association for Palliative Care published guidelines on depression (Rayner et al. 2011).

In view of the frequency of the two symptoms, it seems important to integrate psychiatric and/or psycho-oncologic expertise into PCTs so that these issues can be addressed professionally. The Japanese team composition has a clear advantage here compared to the German situation where psychiatrists or psycho-oncologists are not recommended in a PCT. 82–98% of patients reported family anxiety due to the patient’s situation. This underlines the importance of holistic palliative care for relatives as well as for patients.

Pain is a relevant problem, but not one of the most commonly reported symptoms. In our study, 62–72% of patients reported pain versus 66% of Japanese palliative cancer patients (Sakurai et al. 2019). In the German study of Erlenwein et al. (2014), pain was the explicit reason for consultation with the PCT in 64% of cases, and in the Japanese study of Hatano et al. (2018) pain was the reason for consultation with the PCT in 67% of cases. This is possibly a result of the now improved pain management in general palliative care.

On the other hand, good pain management is in most cases easier than interventions and helps patients to cope with fatigue, weakness or poor mobility. A contemporary review shows that fatigue can be managed with different types of exercise in combination with psycho-education and other non-pharmacological interventions (Hilfiker et al. 2018). The S3-LL Guidelines dedicate a separate chapter to the topic of fatigue (S3-LL, Chapter 10). This shows that the significance of this symptom is now recognised.

### Quality indicator

We used the adapted quality indicator ‘percentage of hospitalised cancer patients with PCT contact 3 days or more before death’ to evaluate the integration of SPC into inpatient end-of-life care. Both PCTs did not achieve the quality goals defined by Earle et al. (2005) of 55% of deceased patients receiving palliative or hospice services (SPC) and less than 8% of them with SPC contact for less than 3 days (PCT-N: 23% of all deceased patients had PCT co-management and 18.3% of all SCP co-managed patients had SPC contact for less than 3 days before death; PCT-W: 33% of all deceased patients had PCT co-management and 39% of PCT co-managed patients had SPC contact for less than 3 days before death). PCT-N seems to have earlier contact with patients, but PCT-W seems to care for more patients.

The thresholds for the quality indicators were expert approved more than 15 years ago. Until now, there has been intensive debate about the right cutoff for quality indicators evaluating SPC integration in hospital and in general. The analysis of Dasch et al. (2017) shows that 30% of deceased cancer patients in a German university hospital had SPC contact, 54% of those cases initiated in the last week of life. In 2018, Sato et al. presented the first retrospective analysis of the administrative data of 248,978 deceased cancer patients in Japanese acute care hospitals regarding end-of-life care. In the last 14 days before death, 8.1% of deceased cancer patients in high-volume hospitals, 2.1% in intermediate-volume hospitals and 2% in low-volume hospitals had PCT contact, meaning that a palliative care additional fee was claimed (Sato et al. 2018). Regarding these two studies, both PCT-N and PCT-W seem to be reasonably integrated into inpatient end-of-life care, although there is room for improvement.

There is an international consensus that inpatient end-of-life care has to be performed by the primary teams together with the palliative care specialists. A structured needs-based approach is recommended because it can provide quality of life for patients more successfully than a consultation-based approach (van Mechelen et al. 2013; Leitlinienprogramm Onkologie 2019, standard operation procedure 5.2). Inpatient screening for complex palliative care needs for patients with incurable and advanced illnesses (cancer) is perhaps an option to close the gap between ambition and reality. The first initiatives have already been described, one of them starting from admission into an emergency department (Weissman and Meier 2011; Glare and Chow 2015; Seekatz et al. 2017; Ostgathe et al. 2019; Reuter et al. 2019), but further research and a broader database are necessary to evaluate this new approach. Until this occurs, the two quality indicators can help to describe the situation and to promote the integration of SPC without rating or benchmarking.

### Limitations and outlook

Our study has several limitations. It is retrospective and cultural aspects as well as aspects of the different health-care systems are neglected. The database is small and a predefined consensus about the documented items is missing. Therefore, only descriptive analysis was performed. Neither PCT-W nor PCT-N document their treatment strategies or patient-reported outcome parameters. Therefore, we used a quality indicator based on administrative data to describe the integration of the two PCTs into inpatient end-of-life care. For a planned prospective joint project, the documentation will first be harmonised, based on national and international standards (Guo et al. 2018; Schoenherr et al. 2019; Nationales Hospiz-und Palliativregister; Leitlinienprogramm Onkologie 2019). The IPOS will also be introduced in PCT-N and in both PCTs a second assessment at the end of the co-management before discharge is planned. The IPOS is responsive to change and has a validated proxy-report version as well as a staff version, so that an assessment near end of life or after death is also possible.

## Conclusion

This exploratory analysis showed that the organisation and work processes of the two PCTs as well as the problems of the patients are similar. In contrast to the public image of palliative care, it is psychosocial needs, not pain, that are mostly cited by patients. In further studies, patient- or relative-reported outcomes should be assessed in addition to administrative data and treatment strategies. An in-depth analysis of cultural aspects and of the different health-care systems should also be part of further comparative analyses.
